# Neuropilin-2 induced by transforming growth factor-β augments migration of hepatocellular carcinoma cells

**DOI:** 10.1186/s12885-015-1919-0

**Published:** 2015-11-16

**Authors:** Philipp Wittmann, Markus Grubinger, Christian Gröger, Heidemarie Huber, Wolfgang Sieghart, Markus Peck-Radosavljevic, Wolfgang Mikulits

**Affiliations:** 1 Department of Medicine I, Division: Institute of Cancer Research, Comprehensive Cancer Center, Medical University of Vienna, Vienna, Austria; 2 Department of Internal Medicine III, Division of Gastroenterology and Hepatology, Medical University of Vienna, Vienna, Austria

**Keywords:** Neuropilin-2, Transforming growth factor-β, Epithelial to mesenchymal transition, Hepatocellular carcinoma

## Abstract

**Background:**

Hepatocellular carcinoma (HCC) is the most common form of liver cancer and the third most lethal cancer worldwide. The epithelial to mesenchymal transition (EMT) describes the transformation of well-differentiated epithelial cells to a de-differentiated phenotype and plays a central role in the invasion and intrahepatic metastasis of HCC cells. Modulation of the transforming growth factor-β (TGF-β) signaling is known to induce various tumor-promoting and EMT-inducing pathways in HCC. The meta-analysis of a panel of EMT gene expression studies revealed that neuropilin 2 (NRP2) is significantly upregulated in cells that have undergone EMT induced by TGF-β. In this study we assessed the functional role of NRP2 in epithelial and mesenchymal-like HCC cells and focused on the molecular interplay between NRP2 and TGF-β/Smad signaling.

**Methods:**

NRP2 expression was analyzed in human HCC cell lines and tissue arrays comprising 133 HCC samples. Cell migration was examined by wound healing and Transwell assays in the presence and absence of siRNA against NRP2. NRP2 and TGF-β signaling were analyzed by Western blotting and confocal immunofluorescence microscopy.

**Results:**

We show that NRP2 is particularly expressed in HCC cell lines with a dedifferentiated, mesenchymal-like phenotype. NRP2 expression is upregulated by the canonical TGF-β/Smad signaling while NRP2 expression has no impact on TGF-β signaling in HCC cells. Reduced expression of NRP2 by knock-down or inhibition of TGF-β signaling resulted in diminished cell migration independently of each other, suggesting that NRP2 fails to collaborate with TGF-β signaling in cell movement. In accordance with these data, elevated levels of NRP2 correlated with a higher tumor grade and less differentiation in a large collection of human HCC specimens.

**Conclusions:**

These data suggest that NRP2 associates with a less differentiated, mesenchymal-like HCC phenotype and that NRP2 plays an important role in tumor cell migration upon TGF-β-dependent HCC progression.

**Electronic supplementary material:**

The online version of this article (doi:10.1186/s12885-015-1919-0) contains supplementary material, which is available to authorized users.

## Background

Liver cancer is the sixth most common cancer in the world and ranks second in the list of most deadly cancers [1]. The vast majority of liver cancers are hepatocellular carcinomas (HCC) representing up to 90 % of all liver malignancies [2, 3]. The main risk factors for HCC are chronic infections with either hepatitis B virus (HBV) or hepatitis C virus (HCV), making up approximately 75–85 % of all cases, as well as excessive alcohol consumption, which is responsible for about 40 % of HCC development in Western countries [2, 4–7]. Chronic inflammation and tissue damage by these agents leads to cirrhosis which is the underlying condition for the majority of HCC cases [8].

The dissemination of primary tumor cells into the body drastically worsens the prognosis of cancer patients [9]. Metastasis of HCC cells most frequently occurs intrahepatically rather than extrahepatically to distal sites such as the lung [10]. For spreading of HCC cells, individual cell movement by an epithelial to mesenchymal transition (EMT) has been considered to be essentially involved [11]. Upon EMT and progression in malignancy, highly differentiated epithelial cells such as hepatocytes de-differentiate into a mesenchymal-like phenotype that exhibits strong migratory abilities. Various signaling cascades are known to induce EMT, such as the Wnt/β-catenin, PI3K/AKT/mTOR, Hedgehog, Ras/Raf/MEK/ERK, Notch and NFκB pathways, as well as transforming growth factor (TGF)-β [12–15]. These signals mostly converge on EMT-transcription factors (EMT-TFs) such as Snail, ZEB1 or Twist1/2 which transcriptionally repress E-cadherin and other epithelial junctional proteins as well as activate a mesenchymal gene expression signature. In HCC, TGF-β signaling has been shown to activate EMT-TFs and to repress their negative feedback loops by the downregulation of miRNAs that antagonize EMT-TFs [16, 17].

A recently performed meta-analysis compared 24 published EMT gene expression data sets and generated a core list of genes that are most frequently altered during EMT [18]. One of the genes found upregulated in several studies of TGF-β-induced EMT coded for the protein neuropilin-2 (NRP2). There are two homologs of the NRP family, NRP1 and NRP2, which are 130 kDa single-pass transmembrane glycoproteins that act as non-tyrosine kinase co-receptors [19]. They contain 4 distinct domains including a CUB domain, a FV/FVIII domain, a MAM domain and a domain that contains the transmembrane and short cytoplasmic region [20]. Both homologues can homo- and heteromultimerize [21] and can bind different members of the semaphorin family, as well as members of the vascular endothelial growth factor (VEGF) family [22]. In addition, NRPs are receptors of hepatocyte growth factor, platelet-derived growth factor BB, fibroblast growth factor, epidermal growth factor, placenta growth factor, and importantly of TGF-β1 [23–25]. NRPs are therefore critical regulators of angiogenesis, lymphangiogenesis and tumor progression. NRP2 expression is correlated with lymph node metastasis in breast cancer and blocking of NRP2 leads to decreased metastasis formation [26–28]. Clinical data show that high NRP levels, in particular NRP2, correlate with poor prognosis and survival in various cancer types [29, 30]. NRP2 was suggested to play a direct role in EMT and a cross-talk between NRP2 and TGF-β1 signaling promotes colorectal cancer progression [31]. In the context of HCC, the role of NRP2 is so far unknown [32].

In this study, we show that NRP2 expression strongly correlates with a mesenchymal phenotype in HCC cell lines and that reduced levels of NRP2 dramatically impair the migratory abilities of HCC cells. We further provide evidence that NRP2 expression is controlled by canonical TGF-β signaling, while no direct impact of NRP2 on TGF-β signaling could be observed. Translation of these data into the HCC patient situation revealed a correlation of NRP2 levels with a poorly differentiated HCC, suggesting a role of NRP2 in TGF-β regulated HCC progression.

## Methods

### Tissue array analysis

Tissue arrays contained paraffin-embedded specimens of tumors and adjacent normal tissue collected from 133 female and male HCC patients. All patients have undergone orthotopic liver transplantation for HCC at the Department of Transplantation Surgery, Medical University of Vienna, between 1982 and 2002, as described [33]. All specimens were reviewed for histological type and grade by 2 individual board certified pathologists. 4 μm thick sections of core biopsies were arrayed in triplicate and stained with anti-NRP2 antibody (R&D Systems, Minneapolis, USA) or anti-TGF-β1 antibody (Santa Cruz, Dallas, USA) at a dilution of 1:100. After incubation with secondary antibodies, visualization was performed using the vectastain ABC system (Vector Laboratories, Burlingame, USA). Triplicates of stained tissues were evaluated by two independent researchers (P.W. and M.G.) who were blinded regarding patient details. Immunostaining for NRP2 was scored by arbitrary scaling of no and low-to-high staining. Immunohistochemical analysis of paraffin-embedded tissues and retrospective analysis of patient data were approved by the ethics committee of the Medical University of Vienna.

### Cell culture

The human hepatoma cell lines 3p, 3sp, SNU-398, SNU-423, SNU-449 and SNU-475 were grown in RPMI-1640 medium plus 10 % fetal calf serum (FCS) and antibiotics. Hep3B, HepG2 and FLC-4 cells were cultivated in Eagle’s Minimum Essential Medium (EMEM) plus 10 % FCS and antibiotics. PLC and HuH-6 cells were cultured in Dulbecco’s Modified Eagle Medium (DMEM) plus 10 % FCS and antibiotics. Human hepatic sinusoidal endothelial cells (HSECs) received endothelial cell medium (Lonza, Basel, Switzerland). All cells were kept at 37 °C and 5 % CO2. TGF-β signaling was stimulated by supplementing the medium with 2.5 ng/mL TGF-β1 (R&D Systems, Minneapolis, USA) for 24 h. For inhibition of TGF-β signaling, LY2109761 (Santa Cruz, Dallas, USA) antagonizing both TGF-β receptors I/II was used at a concentration of 10 μM for 24 h. All HCC cell lines were validated by short tandem repeat analysis.

### siRNA knock-down

Cells were seeded on 6-well plates and either transfected with 80 nM of non-target small interfering (si)RNA or with 80 nM of siRNA against human NRP2 (Dharmacon, Town, UK). Cells were processed for further analysis after 48 h of siRNA transfection.

### Western blot analysis

Immunoblotting was performed as described [34]. Dilutions of primary antibodies were as follows: NRP2 (R&D Systems, Minneapolis, USA), 1:1000; β-actin (Sigma, St. Louis, USA), 1:2500; Smad2/3 (BD Biosciences, NJ, USA), 1:1000; pSmad2 (Upstate, NY, USA), 1:500; Smad4 (Cell Signaling Technology, Danvers, USA), 1:1000. Secondary antibodies conjugated to horseradish peroxidase were used for detection.

### Transwell migration and invasion

2.5 × 10^4^ hepatoma cells were resuspended in 100 μL medium containing 1 % FCS and transferred onto 24-well cell culture inserts with a pore size of 8 μm (Corning, Tewksbury, USA). 600 μL medium containing 10 % FCS was added to lower chambers to generate a gradient for cell migration. Medium was removed and migrated cells on membranes were fixed with 4 % paraformaldehyde after 16 h. Cell nuclei of migrated cells at the bottom side of the membrane were stained with Hoechst 33342 (Life Technologies, Green Island, USA) and counted under the fluorescence microscope (Nikon, Tokyo, Japan). To analyze cell invasion, 24-well cell culture inserts were coated with rat-tail collagen (BD Biosciences, NJ, USA) prior to seeding of hepatoma cells. To examine transendothelial invasion, 2 × 10^5^ HSECs were plated in 100 μL endothelial cell medium (Lonza, Basel, Switzerland) onto coated 24-well cell culture inserts and allowed to form a monolayer for 48 h. Subsequently, endothelial cell medium was aspirated and hepatoma cells were seeded in 100 μL medium containing 1 % FCS onto 24-well inserts. Transmigrated cells were visualized after 16 h and quantified as described for cell migration.

### Wound healing assay

For studying cell migration in a scratch wound assay, cells were seeded in 6-well plates and artificial wounds were inflicted to the cell layer by scratching with sterile pipette tips. For each condition, three scratches were inflicted in three independent wells of a 6-well plate. From each of these scratches eight images were taken for a total of 24 images per condition and time point. Images were performed by phase contrast microscopy (Nikon, Tokyo, Japan) immediately after wounding and after 24 h. The migrated area of cells into the wound was quantified with ImageJ software.

### Quantitative real-time polymerase chain reaction (qPCR)

RNA isolation was performed using an RNeasy purification kit according to the manufacturer’s instructions (Quiagen, Hilden, Germany). For reverse transcription of RNA into DNA, the QuantiTect Reverse Transcription kit was employed (Quiagen, Hilden, Germany). For quantitative RT-PCR (qPCR), aliquots of cDNA were employed for Fast SYBR green qPCR (Applied Biosystems, Foster City, USA) and quantified with the 7500 Fast Real-Time PCR System (Applied Biosystems, Foster City, USA). RPL41 as HCC housekeeping gene and the average ΔC_T_ value of the HCC cell line 3sp were used to calculate the ΔΔC_T_ values for all cell lines. The ΔΔC_T_ values were used to graph the fold-change (RQ) via the formula RQ = 2^(−ΔΔC_T_). Error bars show SE of ΔΔC_T_, calculated with the following formula: SE(ΔΔC_T_) = √((SE(ΔC_T_ Control)^2) ± (SE(ΔC_T_ Target)^2)). The primer sequences are: NRP2 (forward), 5’-CTGTGGGTCATCCGTGAGGAC-3’ and NRP2 (reverse) 5’-ATGGGTTCCATGCAGTTCTCCAG-3’; RPL41 (forward), 5’-CAAGTGGAGGAAGAAGCGA-3’ and RPL41 (reverse), 5’-TTACTTGGACCTCTGCCTC-3’.

### Confocal immunofluorescence microscopy

Cells were seeded on glass cover slips coated with rat-tail collagen (BD Biosciences, NJ, USA) and fixed with 4 % formaldehyde. After permeabilization with 0.25 % Triton-X 100 and blocking with 5 % horse serum, cells were stained with primary antibody against Smad2/3 (BD Biosciences, NJ, USA) at a concentration of 1:100 and further incubated with secondary antibody (1:200), phalloidin (1:750) and DAPI (1:1000). Images were obtained by confocal immunofluorescence microscopy (Zeiss, Oberkochen, Germany).

### Statistical analysis

The statistical significance of differences was evaluated using two-sided Student’s *t*-test. Data are expressed as means ± standard deviation (SD) or means ± standard error of the mean (SEM) for tissue array data.

## Results

### NRP2 is upregulated in high-grade HCC and highly expressed in mesenchymal-like HCC cell lines

**Fig. 1:**
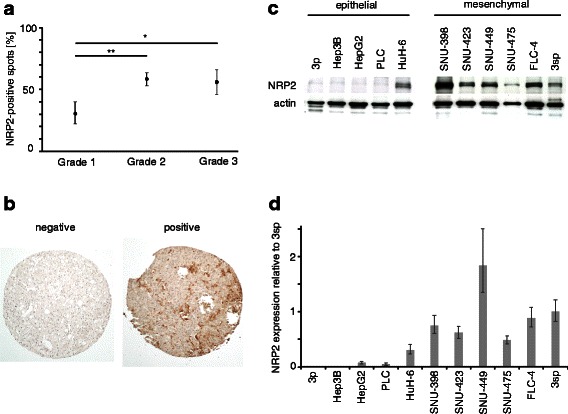
NRP2 expression in primary HCC tissue and HCC cell lines. **a** NRP2 expression correlated with less differentiated HCC of grade 2 and 3. **b** Representative images of no (left) and low-to-high NRP2 expression (right). **c**, **d** Western blot (**c**) and qPCR analysis (**d**) of epithelial and mesenchymal-like HCC cells. Expression of actin is shown as loading control. NRP2 expression in 3sp cells was set to a value of 1 to allow comparison of NRP2 levels in the various cell lines. Error bars depict SD from 3 independent experiments that were performed in triplicates.*, *p* < 0.05; **, *p* < 0.01

We employed a tissue array containing 133 HCC cases for the analysis of NRP2 expression and found a significant correlation of NRP2 levels with higher tumor grading. While well-differentiated HCC (grade 1) showed NRP2 expression in 32 % of cases, less-differentiated HCC samples displayed a nearly twice as much higher frequency of NRP2 presence. In particular, 59 % of HCC with grade 2 and 56 % of HCC with grade 3 tumors showed NRP2 expression (Fig. 1a). Generally, NRP2 was found distributed in a patchy fashion (Fig. 1b) and interestingly, NRP2 was exclusively expressed in those patient samples that showed TGF-β1 expression (Additional file 1: Figure S1). Noteworthy, normal liver did not display NRP2 expression (Fig. 1b) which confirmed recent observations that NRP2 is not expressed in hepatocytes under physiological conditions [35].

To correlate NRP2 expression with differentiation of hepatoma cells more closely, we analyzed various human HCC cell lines with differentiated and epithelial traits versus those exhibiting a de-differentiated and mesenchymal-like phenotype. Western blot (Fig. 1c) and qPCR analyses (Fig. 1d) revealed that NRP2 expression strongly correlated with a mesenchymal-like HCC phenotype in 3sp, SNU-398, SNU-423, SNU-449, SNU-475 and FLC-4 cells. In contrast, NRP2 was expressed in only one out of five epithelial HCC cell lines, i.e., HuH-6 cells, and showed undetectable levels of NRP2 in the other epithelial 3p, Hep3B, HepG2 and PLC cells. Together, these data suggest that NRP2 expression associates with less-differentiated high-grade tumors and TGF-β1 expression in HCC patients. In agreement, NRP2 expression was found in de-differentiated mesenchymal-like HCC cells in vitro which supports the idea that NRP2 expression correlates with a de-differentiated phenotype.

### NRP2 regulates HCC cell migration and invasion

**Fig. 2:**
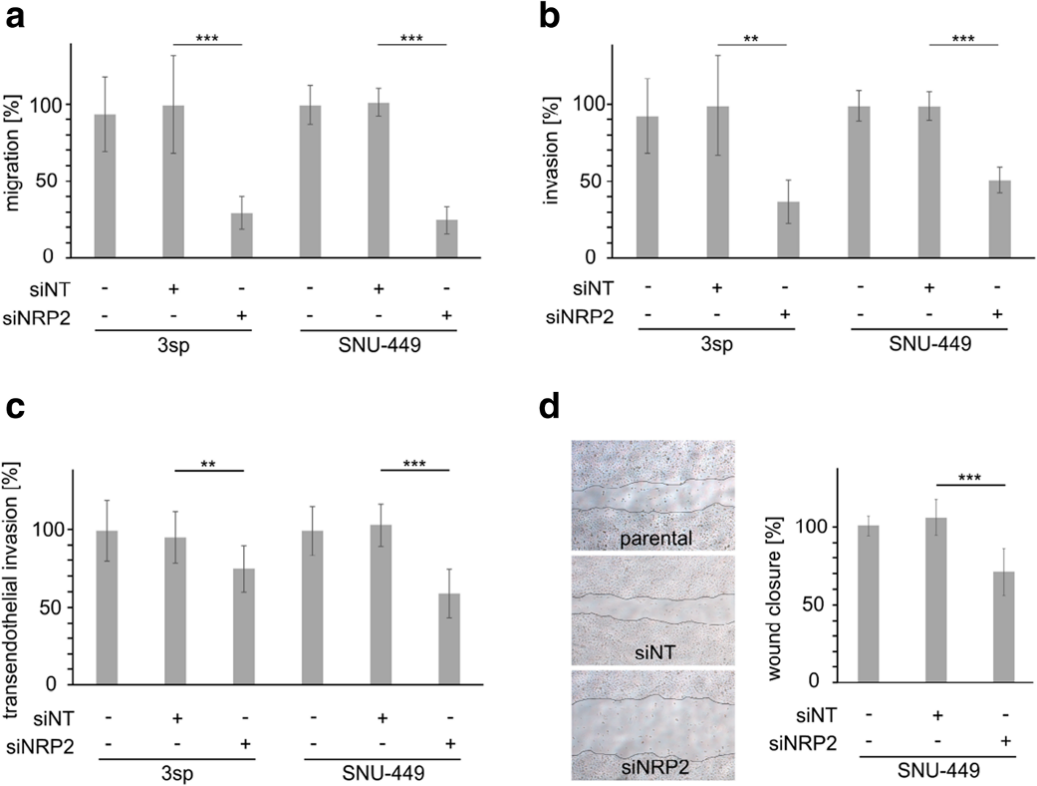
Loss of NRP2 impairs migration of HCC cells. **a** Transwell migration of 3sp and SNU-449 cells either untreated or treated with non-target siRNA (siNT) or siRNA against NRP2 (siNRP2). **b**, **c**Transwell invasion (**b**) and transendothelial invasion (**c**). **d** Migration of SNU-449 analyzed by wound healing assays. Images show migration of cells after 24 h (left panel). Quantification of migrated cells (right panel). The migration of untreated parental SNU-449 cells was set to 100 %. Error bars depict SD from 3 independent experiments that were carried out in triplicates. **, *p* < 0.01; ***, *p* < 0.001

Next we analyzed the functional impact of NRP2 expression on cell proliferation and migration. Neither knock-down nor exogenous overexpression of NRP2 significantly affected proliferation kinetics of mesenchymal-like HCC cells (data not shown). However, Transwell migration assays employing the mesenchymal-like cell lines 3sp and SNU-449 revealed that reduced expression of NRP2 results in impaired migratory abilities (Fig. 2a). Comparable results were obtained in Transwell invasion and transendothelial migration assays (Fig. 2b, c). In addition, assessment of cell migration by wound healing assays of SNU-449 cells confirmed elevated migratory capabilities dependent on NRP2 (Fig. 2d). Interestingly, overexpression of NRP2 could not enhance migration or invasion in any of the employed cell lines (3p, PLC, 3sp, SNU-449; data not shown). Thus, these results suggest that NRP2 is crucially involved in upregulating cell movement of de-differentiated HCC cells.

### TGF-β induces NRP2 in a Smad-dependent fashion

**Fig. 3:**
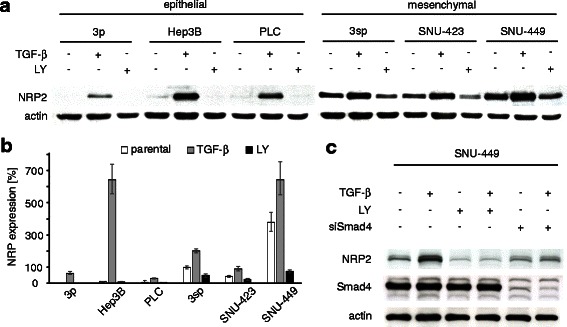
NRP2 expression depends on TGF-β/Smad signaling. **a** NRP2 protein expression after stimulation with 2.5 ng/mL TGF-β1 for 24 h as determined by Western blotting. Actin is shown as loading control. **b** qPCR analysis showing NRP2 mRNA levels in epithelial and mesenchymal-like HCC cell lines treated with either 2.5 ng/mL TGF-β1 or with 10 μM TGF-β inhibitor LY2109761 (LY) for 24 h. **c** SNU-449 hepatoma cells treated with control siRNA (siNT) and either administrated with 2.5 ng/mL TGF-β or 10 μM LY2109761 (LY) alone or in combination and analyzed for NRP2 and Smad4 expression by Western blotting. Smad4 knock-down (siSmad4) of SNU-449 cells treated with 2.5 ng/mL TGF-β1 for 24 h did not show modulation of NRP2 levels. Error bars depict SD from 3 independent experiments that were performed in triplicates

Since NRP2 expression correlated with TGF-β1 expression (Additional file 1: Figure S1) and TGF-β signaling is a common trigger of EMT in HCC, we asked whether it directly influences NRP2 expression in HCC cells. Remarkably, TGF-β treatment of epithelial 3p, Hep3B and PLC cells, as well as mesenchymal-like 3sp, SNU-423 and SNU-449 cells resulted in increased NRP2 protein expression (Fig. 3a). Upregulation of NRP2 was essentially confirmed at transcript levels by qPCR analysis of epithelial (3p, Hep3B, PLC) and mesenchymal-like HCC cells (3sp, SNU-423, SNU-449) (Fig. 3b). Interestingly, blocking of TGF-β signaling by the TGF-β receptor inhibitor LY2109761 resulted in downregulation of NRP2 mRNA and protein to levels even lower than those of untreated cells (Fig. 3a–c), suggesting autoregulatory TGF-β loops in respective HCC cells. Importantly, cells with interference of canonical TGF-β/Smad signaling by knock-down of Smad4 were insensitive to TGF-β-induced upregulation of NRP2 (Fig. 3c). These data indicate that augmented NRP2 expression is caused by canonical TGF-β/Smad signaling.

### TGF-β-independent activity of NRP2

**Fig. 4:**
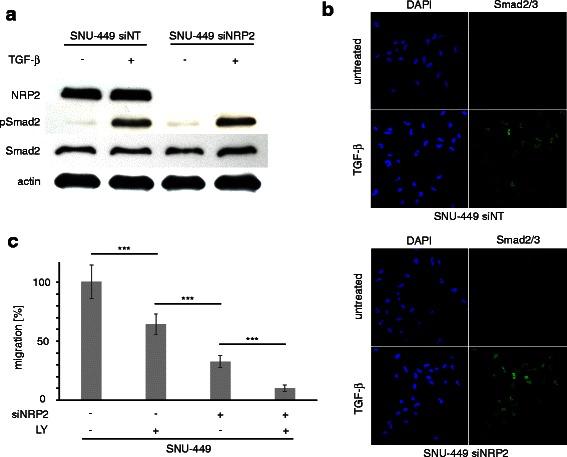
Effects of NRP2 on TGF-β Signaling and synergistic effects on migration. **a** Knock-down of NRP2 does not affect phosphorylation of Smad2 (pSmad2) in SNU-449 cells after treatment with 2.5 ng/mL TGF-β1 for 6 h. Actin was used as loading control. **b** Translocation of Smad2/3 into the nucleus of SNU-449 cells treated with control siRNA (siNT) or siRNA against NRP2 (siNRP2) after treatment with 2.5 ng/mL TGF-β1 for 1 h. **c** Transwell assays of SNU-449 cells assessing the migratory impact of reduced TGF-β signaling by LY2109761 (LY) and diminished NRP2 expression by knock-down (siNRP2), either alone or in combination. The treatment of cells with non-target siRNA (siNT) was set to 100 %. Error bars depict SD from 3 independent experiments that were carried out in triplicates. ***, *p* < 0.001

We next examined whether NRP2 affects canonical TGF-β signaling in a feedback loop. First, we analyzed the level of phosphorylated Smad2 protein that displays TGF-β signaling in SNU-449 cells with and without a NRP2 knock-down after treatment with TGF-β. No difference in pSmad2 levels were observed, independently whether or not NRP2 was expressed (Fig. 4a). To confirm these data, we performed confocal immunofluorescence analysis of TGF-β-treated NRP2 knock-down cells and analyzed nuclear Smad2/3 staining (Fig. 4b). In accordance, no differences in nuclear Smad2/3 localization between control and NRP2 knock-down cells were detected, suggesting that NRP2 does not influence the canonical TGF-β/Smad signaling.

Interference with TGF-β signaling also leads to a reduced migration of mesenchymal-like HCC cells. Hence, we further analyzed whether the effect of reduced NRP2 on cell migration is dependent on TGF-β signaling or not by employing NRP2 knock-down and control cells after treatment with TGF-β inhibitor LY2109761 (Fig. 4c). Cells treated with LY2109761 reached approximately 64 % migration efficiency compared to control siNT cells. NRP2 knock-down cells without inhibitor treatment were even slower migrating, reaching about 33 % of cell motility. Importantly, the cells treated with both siRNA against NRP2 and LY2109761 migrated even significantly slower than those treated with LY2109761 only, reaching about 10 % compared to reference. In conclusion, these findings indicate that NRP2 has a role in cell migration that is independent of TGF-β signaling.

## Discussion

Of the two neuropilin isoforms, NRP1 is the far more examined protein. Its role in various types of cancer is well documented and often linked to angiogenesis via VEGF. In contrast, few studies were dedicated to NRP2 and its role in tumorigenesis. To our knowledge no attempts were made to elucidate its role in HCC.

In this study, we showed that NRP2 expression correlates with less differentiation in HCC patients as well as with a de-differentiated, mesenchymal-like phenotype of HCC cell lines (Fig. 1). Functionally, NRP2 expression severely enhances migration of mesenchymal-like HCC cells and is induced by the canonical TGF-β/Smad signaling. In this setting, NRP2 can be considered as a biomarker of both the activation of TGF-β/Smad signaling and loss of differentiation by EMT. Thus, NRP2 represents a novel marker for EMT-transformed cells that can be used apart from the classical ones such as increased vimentin and N-cadherin or loss of ZO-1 and E-cadherin expression [36].

Diminished migration of HCC cells was similar either after reducing NRP2 levels or after blocking TGF-β signaling (Fig. 4c). TGF-β inhibition on its own reduced NRP2 expression but could not completely block it (Fig. 3a, b), as the remaining NRP2 levels might allow cells to better migrate as compared to those cells with strongly reduced NRP2 levels after RNA interference. Yet, if HCC cells were blocked for both NRP2 and TGF-β signaling, a further decrease in HCC cell migration was observed (Fig. 4c). This additive operation of NRP2 and TGF-β signaling suggests that TGF-β additionally affects cell migration that is independent of NRP2. Moreover, neither overexpression of exogenous NRP2 nor treatment with TGF-β1 had any detectable positive effect on migration (data not shown), suggesting that neither NRP2 expression nor TGF-β signaling are rate limiting in migration of mesenchymal-like HCC cell lines. In addition, the consequences of NRP2 expression on migration and invasion as determined by the passing of cells through unchanged or collagen-coated Transwell membranes was stronger than in follow-up experiments in which cells were allowed to transmigrate through a layer of endothelial cells. This suggests that NRP2 mainly affects the migratory potential of HCC cells rather than their ability of breaking through endothelial barriers.

We could show for the first time that NRP2 expression is tightly controlled by the canonical Smad2/3-Smad4 signaling cascade in HCC cells (Fig. 3c). NRP2 is therefore considered as an effector of active TGF-β signaling. Interference with TGF-β signaling decreases NRP2 expression below levels of untreated cells (Fig. 3a), indicating autocrine regulatory TGF-β signaling in mesenchymal-like HCC cells [37]. In this line, NRP2 expression could be used as marker of persistent TGF-β activity in HCC patients which is indicative of HCC progression via the tumor promoting arm of TGF-β [38, 39]. This could explain why NRP2 expression is associated with higher grading in HCC specimens (Fig. 1a). Interestingly, no other significant correlations between NRP2 expression and e.g., tumor staging or vessel invasion could be found which might be due to limitations in the immunohistochemical analysis of tissue arrays.

TGF-β is molecularly linked to NRP2 expression as TGF-β induces NRP2 levels (Fig. 3a, b). However, we found no impact of NRP2 on canonical TGF-β signaling (Fig. 4a, b) despite the fact that NRP2 has been described to act as TGF-β co-receptor [31]. Interestingly, a role of NRP2 in canonical TGF-β signaling could also not be confirmed in lung cancer cells [40], however, this study showed that NRP2 affected ERK signaling supposedly via non-canonical TGF-β signaling activity. In HCC cells, we could also not detect NRP2-driven non-canonical TGF-β signaling (data not shown). The question how NRP2 exerts its influence on cell migration remains open. NRP2 could act on other signaling pathways controlling migration or NRP2 could directly act as a receptor that conducts signals via its intracellular domain. These aspects are the basis for further investigations and allow assessing whether NRP2 represents a valuable target for HCC intervention.

## Conclusions

TGF-β shows anti-proliferative and tumor-suppressing functions in healthy liver tissue but upon HCC development, aberrant TGF-β signaling is a major driver of HCC progression. We found that NRP2 is induced by canonical TGF-β/Smad signaling in HCC cells and that NRP2 is a potent regulator of HCC cell migration and invasion. NRP2 associates with a mesenchymal-like phenotype in vitro and accordingly, NRP2 correlates with a higher tumor grade in vivo indicating less differentiation. NRP2 is therefore thought to play an important role in TGF-β-dependent HCC progression.

## Additional file

Additional file 1: Figure S1. Correlation of NRP2 expression and TGF-β1 expression in vivo. The tissue array comprising 133 HCC patients was analyzed for TGF-β1 expression and correlated with NRP2 levels. TGF-β1 expression was scored by arbitrary scaling of no, low, medium and high staining as described [37]. (PDF 34 kb)

## Abbreviations

EMT: Epithelial to mesenchymal transition
HCC: Hepatocellular carcinoma
NRP2: Neuropilin-2
qPCR: Quantitative reverse-transcription polymerase chain reaction
siRNA: Small interfering RNA
TGF-β: Transforming growth factor-β
