# Supplementary figures and images for: Neuropilin-2 induced by transforming growth factor-β augments migration of hepatocellular carcinoma cells
Source: BMC Cancer. 2015 Nov 16;15:909. doi: 10.1186/s12885-015-1919-0 (PMC4647494; doi:10.1186/s12885-015-1919-0)

Supplementary Figure 1; Wittman et al.

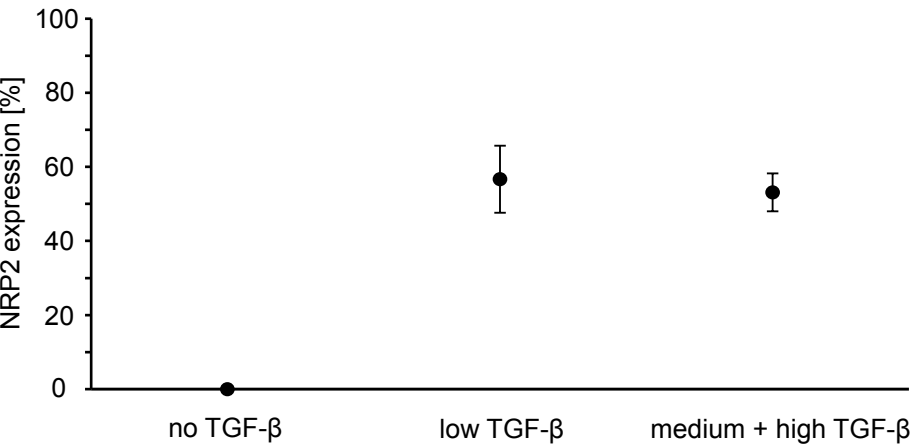

Supplement: Additional file 1: Figure S1. — Correlation of NRP2 expression and TGF-β1 expression in vivo. The tissue array comprising 133 HCC patients was analyzed for TGF-β1 expression and correlated with NRP2 levels. TGF-β1 expression was scored by arbitrary scaling of no, low, medium and high staining as described [37]. (PDF 34 kb) [file 12885_2015_1919_MOESM1_ESM.pdf]
